# Ruminal microbial dysbiosis induces mastitis in dairy goats by activating oxidative stress and ferritinophagy-ferroptosis

**DOI:** 10.1016/j.isci.2025.114012

**Published:** 2025-11-12

**Authors:** Yuhong He, Wei Zhang, Can Zhang, Nier Su, Zeming Zhou, Chong Peng, Chongshan Yuan, Yunhe Fu, Xiaoyu Hu, Yue Zhang

**Affiliations:** 1 Department of Anesthesiology, China-Japan Union Hospital of Jilin University, Erdao District, 126 Sendai Street, Changchun, Jilin Province 130033, China; 2 Department of Clinical Veterinary Medicine, College of Veterinary Medicine, Jilin University, Changchun, Jilin Province 130062, China; 3 College of Animal Science and Technology, Jilin Agriculture Science and Technology University, Jilin City, China

**Keywords:** Microbiology, Cell biology

## Abstract

Recent studies indicate that ruminal microbiota dysbiosis plays a significant role in the development of mastitis in ruminants, though the exact mechanisms remain incompletely understood. In this study, a high-concentration (HC) diet led to subacute ruminal acidosis (SARA) in dairy goats, as evidenced by prolonged daily periods of ruminal pH below 5.8. The HC group exhibited disrupted ruminal microbiota, elevated serum levels of LPS, TNF-α, and IL-1β, and a markedly increased milk somatic cell count (SCC) exceeding 500,000/mL, indicating mastitis. Ruminal microbiota transplantation (RMT) from HC goats induced mastitis in mice, which is associated with oxidative stress and ferritinophagy-ferroptosis. The administration of ferrostatin-1 (Fer-1) alleviated mastitis in mice. Further *in vitro* experiments showed that LPS dose-dependently triggered oxidative stress and ferritinophagy-ferroptosis in mouse mammary epithelial cells (MMECs). Collectively, HC diet-induced ruminal dysbiosis elevates systemic and mammary LPS levels, reduces antioxidant capacity, and activates oxidative stress and ferritinophagy-ferroptosis, ultimately leading to mastitis.

## Highlights

• HC diet induces SARA, rumen microbiota dysbiosis, and mastitis in dairy goats
• HC diet induces mammary oxidative stress and ferritinophagy-ferroptosis
• RMT from the HC group to mice induces mastitis, rather than the LC group
• LPS induces oxidative stress and ferritinophagy-ferroptosis in MMECs

## Graphical abstract

**Figure:**
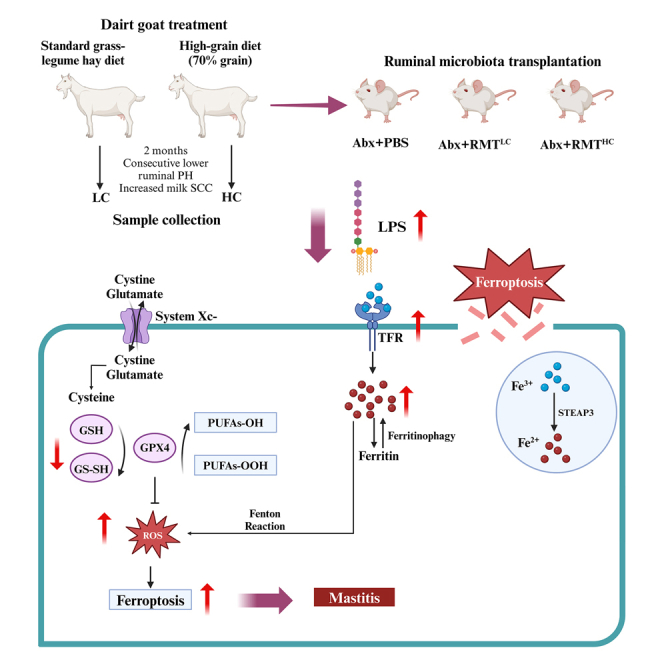

## Introduction

Mastitis represents a major infectious disease affecting dairy goats worldwide, exerting detrimental effects on milk yield and composition while posing significant challenges to dairy productivity and animal health. It not only results in significant economic losses for the farming industry but also poses a heightened risk to food safety associated with dairy products.^1^^,^^2^ The factors that give rise to the development of mastitis in dairy goats are comparable to those observed in other ruminant species. Among these factors are improper feeding practices, infection by pathogenic microorganisms, and external environmental influences.^3^ Notably, in addition to the common pathogenic microorganisms *Staphylococcus aureus*,^4^ *Streptococcus agalactiae*,^5^ and *Escherichia coli*,^6^ which infect ruminants to induce mastitis, several studies have demonstrated that prolonged feeding of an HC diet-induced SARA induces mastitis as well.^7^^,^^8^ In response to the growing demand for milk products in recent years, farmers have frequently supplemented the diet of dairy goats with concentrate content so as to increase milk production.^9^ However, the long-term feeding of HC diet has been shown to result in the accumulation of organic acids in the rumen, which in turn leads to a decline in rumen buffering capacity. Consequently, there is a reduction in rumen pH and an associated increase in the incidence of SARA.^10^ The occurrence of SARA is dependent on the pH of the rumen fluid, which is influenced by the method of collection. Generally, the pH of rumen fluid obtained through rumen puncture, rumen fistula, and oral probe should be below 5.5, 5.8, and 5.8, respectively, to confirm the presence of SARA.^10^ Previous studies have demonstrated that SARA impacts the health and production performance of dairy goats to a significant degree. For example, it affects feed intake, milk production, rumen microbiota, and other functions.^11^ It also induces a series of metabolic diseases, including laminitis, mastitis, and liver abscess.^12^ The existing literature indicates that SARA elevates LPS concentrations in rumen fluid, mammary gland, and plasma of dairy cows, thereby inducing mastitis or increasing susceptibility to pathogen-induced mastitis.^13^

Oxidative stress is a phenomenon whereby organisms produce a higher quantity of oxygen-free radicals than their antioxidant systems are able to scavenge, which results in damage to cells and tissues.^14^ As such, oxidative stress is closely associated with the development of numerous diseases. Oxidative stress occurs when there is an imbalance between the overproduction of reactive oxygen species (ROS) and the antioxidant defense system.^14^ Indeed, oxidative stress plays a role in the development of various diseases, including ketosis, fatty liver, hypocalcemia and mastitis in dairy cows, through its involvement in the body’s amino acid and glucose metabolism.^15^ Interestingly, long-term feeding of HC diets has been demonstrated to induce oxidative stress by increasing peripheral blood LPS levels, which in turn results in a reduction in the antioxidant capacity of mammary tissue.^16^ The concept of “ferroptosis” was proposed in 2012 as a novel and distinctive form of cell death.^17^ It is driven by iron-dependent phospholipid peroxidation and regulated by multiple cellular metabolic pathways, including those governing redox homeostasis, iron metabolism, mitochondrial activity and amino acid, lipid and sugar metabolism.^18^ The principal features of ferroptosis are mitochondrial atrophy, iron accumulation, lipid peroxidation, reduced glutathione (GSH) content and reduced glutathione peroxidase 4 (GPX4) expression.^19^ It is noteworthy that oxidative stress induces cardiomyocyte ferroptosis via mechanisms dependent on GPX4 and Bach1/HO-1.^20^ Moreover, the extant literature has demonstrated that ferroptosis is a pivotal factor in the pathogenesis of mastitis.^21^ Nevertheless, the role of ferroptosis in the development of mastitis in dairy goats resulting from the prolonged administration of HC diets remains to be elucidated.

Consequently, the objective of this study was to ascertain whether oxidative stress and ferroptosis play a role and elucidate their mechanism in mastitis induced by long-term feeding of HC diets in dairy goats. Our findings suggest that the pathogenesis of mastitis in the HC group of dairy goats with disrupted rumen microbiota may be associated with oxidative stress and ferroptosis, as evidenced by significantly elevated rumen, plasma, and mammary levels of LPS. Furthermore, the RMT from the HC group, but not the LC group, significantly induced mastitis and ferroptosis in mice. Subsequently, we demonstrated that elevated endogenous LPS from disrupted rumen microbiota serves as a critical factor that instigates oxidative stress and ferroptosis, which exhibits a dose-dependent capacity to induce oxidative stress and ferroptosis in MMEC. In conclusion, the results of this study indicate that an HC diet induces mastitis in dairy goats by disrupting the rumen microbiota, increasing LPS levels in plasma and mammary tissues, and consequently reducing the total antioxidant capacity and inducing oxidative stress and ferroptosis.

## Results

### High-concentrate diet induces mastitis in dairy goats

**Figure 1:**
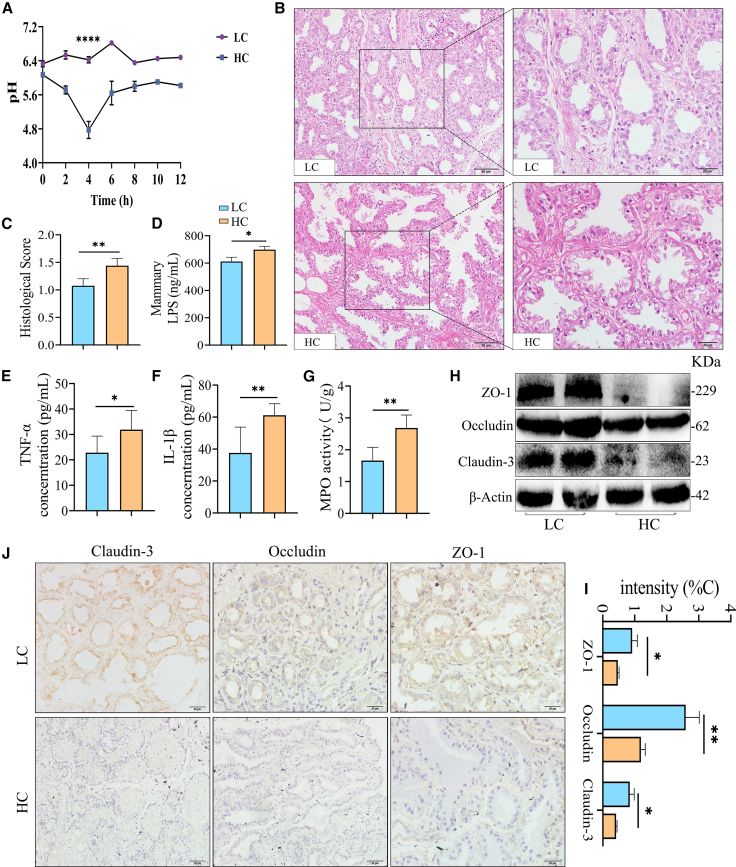
High-concentrate diet induces mastitis in dairy goats (A) Detection of rumen fluid pH in the control group and SARA dairy goats induced by HC diet. (B) Representative H&E-stained images of mammary tissues from dairy goats in LC and HC groups (scale bars, 50 μm). (C) Histopathological score of the mammary gland (*n* = 6). (D–G) The concentrations of LPS (D), TNF-α (E), and IL-1β (F), and MPO activity (G) in mammary tissues of dairy goats in the LC and HC groups were determined using kits (*n* = 6). (H–I) The expression levels of the TJ proteins ZO-1, Occludin, and Claudin-3 in representative mammary tissues were determined by western blotting (H), and the relative intensity of the three proteins was quantified using β-actin as an internal reference (I) (*n* = 3). (J) The localization and expression of TJ proteins in the mammary gland were determined by immunohistochemistry (scale bars, 20 μm). Data are presented as the means ± SD, and one-way analysis of variance (ANOVA) was performed for statistical analysis. ∗*p* < 0.05, ∗∗*p* < 0.01, ∗∗∗*p* < 0.001, and ∗∗∗∗*p* < 0.0001 indicate significant differences. See also Figure S1.

To evaluate the impact of long-term feeding of HC diets on mastitis in dairy goats, we separated the goats into LC and HC groups and fed them the corresponding diets for 8 weeks. As shown in Figure 1A, the rumen pH of goats was significantly reduced, with the rumen pH falling below 5.8 for more than 3 h each day, which indicated that the HC diet successfully established a SARA model. In the mammary glands of the HC group, H&E staining revealed mammary epithelial damage with moderate inflammatory cell infiltration (Figures 1B and 1C). Additionally, SCC in the milk of dairy goats in the HC group was significantly higher than that in the LC group (Figure S1A). Secondly, as has been demonstrated in previous research, low pH in the rumen drives gram-negative bacteria to lyse and release enormous amounts of LPS, and that high rumen acidity and osmotic pressure damage the barrier function of the rumen epithelium, allowing LPS to be released into the blood.^22^^,^^23^^,^^24^ Consistent with earlier research, LPS levels were considerably higher in mammary tissues, rumen tissue, and serum of goat in the HC group (Figures 1D, S1B, and S1C). Additionally, the concentrations of pro-inflammatory cytokines TNF-α and IL-1β were significantly elevated in mammary tissues and serum of HC goats compared to the LC group (Figures 1E, 1F, and S1D–S1E). Hematological examinations revealed that the dairy goats in the HC group exhibited notable hematological and biochemical characteristics, including elevated alkaline phosphatase, albumin, leukocytes4, and percentage of neutrophils, in comparison to the LC group (Figures S1F and S1G). MPO is a marker of neutrophil function and activation. Our results demonstrated that MPO activity was significantly elevated in mammary gland tissues of dairy goats in the HC group (Figure 1G). Tight junction proteins (TJs) between epithelial cells are key determinants of the permeability and structural integrity of the blood-milk barrier (BMB). The results of the western blot and IHC analyses demonstrated a significant reduction in the expression of TJs, including ZO-1, Occludin, and Claudin-3, in the mammary glands of dairy goats in the HC group (Figures 1H–1J). Taken together, our findings indicate that long-term feeding of HC diets results in the disruption of the BMB, the occurrence of mastitis, and the development of systemic inflammation in dairy goats.

### High-concentrate diets induce rumen microbiota dysbiosis in dairy goats

**Figure 2:**
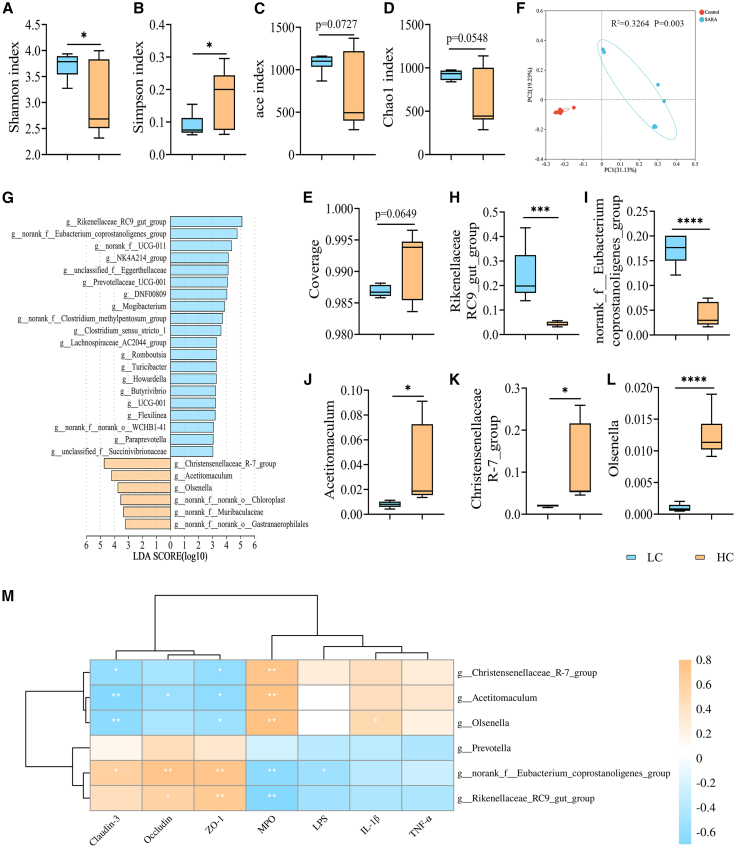
High-concentrate diets induce rumen microbiota dysbiosis in dairy goats (A–E) The alpha diversity indices (including Shannon (A), Simpson (B), ace (C), Chao 1 (D) indices, and Coverage (E)) showed that HC diets reduced the diversity of the rumen microbiota. (F) The PCoA revealed a clear structural separation of the ruminal microbiota between the LC and HC groups of dairy goats, as indicated by the unweighted Unifrac distance (R^2^ = 0.3264, *p* = 0.003). (G) Different bacterial taxa were revealed by LEfSe to be enriched in various groups (log10 LDA score >5). (H–L) Relative abundances of identified different genera in the rumen microbiota. (M) Spearman correlation analysis was used to determine the relationship between important rumen bacterial taxa and inflammatory markers. Yellow denotes a positive correlation; blue denotes a negative correlation. The strength of the Spearman correlation is inversely correlated with the color’s saturation. Data are expressed as a boxplot. ∗*p* < 0.05, ∗∗*p* < 0.01 and ∗∗∗*p* < 0.001 by Mann-Whitney U test. See also Figure S2.

It is of paramount importance to maintain ruminal microecological balance in order to ensure the health of ruminants.^25^ However, sustained low pH in the rumen can lead to the disruption of microecological balance.^26^ Subsequently, the alterations in the rumen microbiota of dairy goats in the HC group were further investigated through 16S rRNA gene sequencing. The results demonstrated that the diversity index Shannon of the rumen microbiota of dairy goats in the HC group was reduced in comparison to that of the LC group, while the Simpson index was elevated (Figures 2A and 2B). Furthermore, no significant difference was observed in the Chao1, ace index, and Coverage indices (Figures 2C–2E). Venn analysis demonstrated a significant difference in the composition of the rumen microbiota between the two groups, with a total of 648 core species co-occurring (Figure S2A). The principal coordinate analysis (PCoA) at the operational taxonomic unit (OTU) level revealed a significant difference in the composition of the rumen microbiota between the two groups (R^2^ = 0.3264, *p* = 0.003, Figure 2F). In particular, the abundance of the *Firmicutes* and *Bacteroidota* decreased, and that of *Actinobacteriota* increased in the rumen microbiota of dairy goats in the HC group compared to the LC group (Figure S2B). At the genus level, there was a relative decrease in the abundance of *Rikenellaceae_RC9_gut_group*, *norank_f Eubacterium_coprostanoligenes_group,* and an increase in the abundance of *norank_f_Bifidobacteriaceae* in dairy goats in the HC group (Figure S2C). To determine the difference in the degree of bacterial enrichment between the LC and HC groups, we performed a linear discriminant analysis effect size (LEfSe) based on LDA scores >5 (Figure 2G). The results demonstrated that the abundance of *Rikenellaceae_RC9_gut_group* and *norank_f Eubacterium_coprostanoligenes_group* significantly decreased in the HC group in comparison to the LC group (Figures 2H and 2I). Conversely, the abundance of *Acetitomaculum*, *Christensenellaceae_R-7_group,* and *Olsenella* increased (Figures 2J–2L). Moreover, the Spearman correlation analysis revealed that the enriched *Acetitomaculum*, *Christensenellaceae_R-7_group,* and *Olsenella* in the HC group exhibited a positive correlation with indicators of inflammation of the mammary glands and a negative correlation with indicators of the BMB (Figure 2M).

### Ruminal microbiota transplantation from high-concentrate dairy goats to mice induces mastitis and disrupts the blood-milk barrier in mice

**Figure 3:**
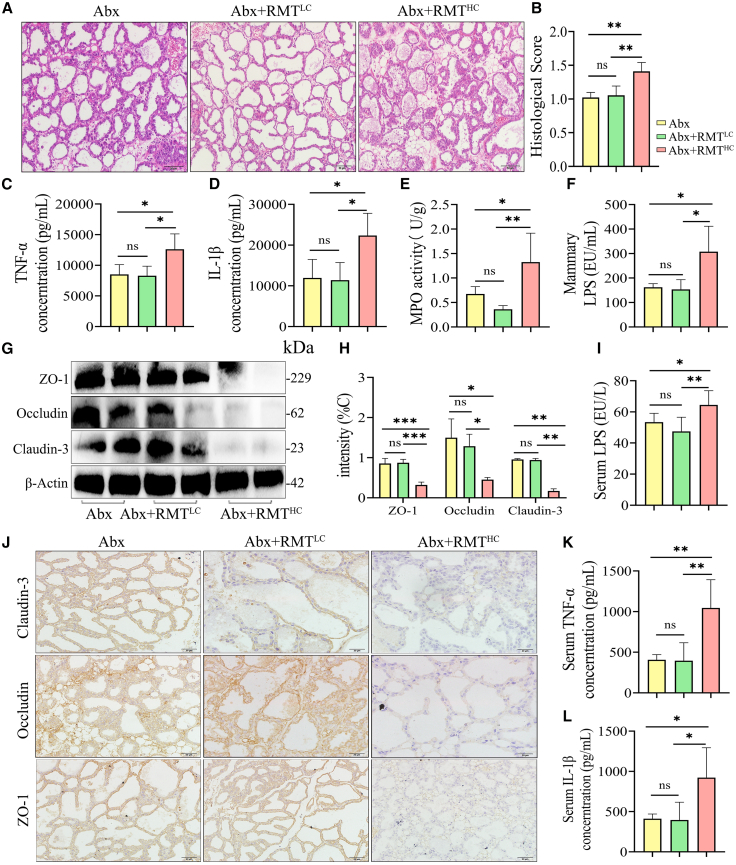
RMT from high-concentrate dairy goats to mice induces mastitis and disrupts the blood-milk barrier in mice (A) Representative H&E images of mammary glands in groups Abx, Abx+RMT^LC^, and Abx+RMT^HC^ (scale bars, 50 μm). (B) Histological score of the mammary gland (*n* = 6). (C–F) Concentrations of TNF-α (C), and IL-1β (D), MPO activity (E), and LPS (F) levels in the mammary gland (*n* = 6). (G and H) Representative images and relative intensities of ZO-1, Occludin, and Claudin-3 in mammary glands (*n* = 3). (I) Serum LPS concentration in groups Abx, Abx+RMT^LC^, and Abx+RMT^HC^ (*n* = 6). (J) The localization and expression of TJ proteins in the mammary gland were determined by immunohistochemistry (scale bars, 20 μm). (K and L) Concentrations of TNF-α (K) and IL-1β (L) in the serum of mice in groups Abx, Abx+RMT^LC^, and Abx+RMT^HC^. Data are presented as the means ± SD, and one-way analysis of variance (ANOVA) was performed for statistical analysis. ∗*p* < 0.05, ∗∗*p* < 0.01, ∗∗∗*p* < 0.001, and ∗∗∗∗*p* < 0.0001 indicate significant differences.

To further confirm the causal relationship between rumen microbiota dysbiosis and the development of mastitis, rumen microbiota from dairy goats in the LC and HC groups were transplanted into antibiotic-pretreated mice. The results demonstrated that mice in the Abx+RMT^HC^ group had inflammatory cell infiltration, the disruption of mammary acinar structure, and elevated inflammatory scores in the mammary glands compared to the Abx group and the Abx+RMT^LC^ group (Figures 3A and 3B). Abx+RMT^HC^ increased the concentration of inflammatory cytokines TNF-α, IL-1β, and MPO activity in mammary tissues compared to the Abx or Abx+RMT^LC^ groups (Figures 3C–3E). Notably, the mammary LPS level was considerably higher in the Abx+RMT^HC^ group of mice (Figure 3F), and the western blot assay revealed a decrease in the expression of TJs proteins, including ZO-1, Occludin, and Claudin-3, in the mammary tissues of mice in the Abx+RMT^HC^ group, which was confirmed by IHC (Figures 3G, 3H, and 3J). In addition, serum LPS, TNF-α, and IL-1β levels were higher in the Abx+RMT^HC^ group compared to the Abx group or the Abx+RMT^LC^ group (Figures 3K, 3L, and 3I), indicating that the transplantation of the rumen microbiota of dairy goats in the HC group induced systemic inflammatory responses in mice. In conclusion, these findings indicate that Abx+RMT^HC^, but not Abx+RMT^LC^, induces mastitis and systemic inflammatory responses in mice, disturbing the BMB.

### High concentrate diets induce oxidative stress and ferroptosis in the mammary tissue of dairy goats

**Figure 4:**
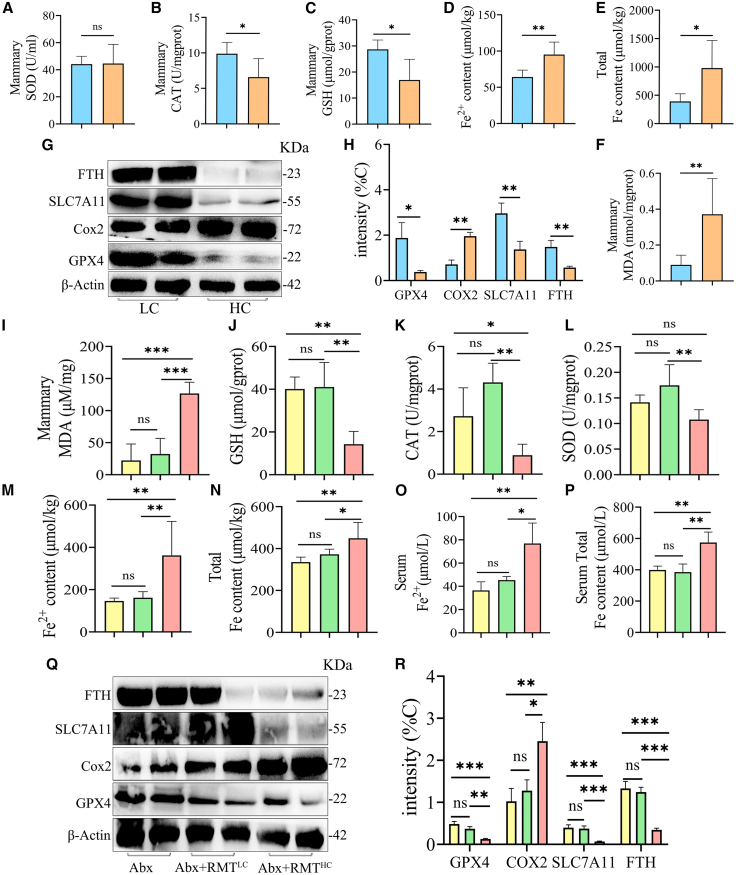
High concentrate diets induce oxidative stress and ferroptosis in the mammary tissue of dairy goats (A) SOD enzyme activity in mammary tissue of the LC group and HC dairy goats. (B) CAT enzyme activity in mammary tissue of the LC group and HC dairy goats. (C) GSH level was measured in different groups using GSH Assay Kit. (D and E) Fe^2+^ (D) and total iron (E) content of mammary tissue in both groups. (F) Measurement of MDA in mammary tissue using an MDA assay kit. (G and H) Representative images and relative intensities of ferroptosis-associated proteins in mammary tissue of dairy goats in LC and HC groups (*n* = 3). (I–N) MDA level (I), GSH content (J), enzyme activity of CAT (K), enzyme activity of SOD (L), and Fe^2+^ (M) and total iron (N) content in mammary tissue of mice in groups Abx, Abx+RMT^LC^, and Abx+RMT^HC^. (O and P) Serum levels of Fe^2+^ and total iron in three groups of mice. (Q and R) Representative images and relative intensities of ferroptosis-associated proteins in mammary tissue from three groups of mice (*n* = 3). Data are presented as the means ± SD, and one-way analysis of variance (ANOVA) was performed for statistical analysis. ∗*p* < 0.05, ∗∗*p* < 0.01, ∗∗∗*p* < 0.001, and ∗∗∗∗*p* < 0.0001 indicate significant differences.

Ma et al. demonstrated that HC diets impair the antioxidant capacity of mammary tissue, induce oxidative stress, and inhibit mammary lipid synthesis in dairy cows.^27^ Furthermore, ferroptosis is a key factor in the pathogenesis of clinical mastitis in dairy cows,^21^ with numerous studies indicating that oxidative stress induces ferroptosis by promoting lipid peroxidation through GSH depletion and GPX4 inactivation.^20^ However, no study has yet reported whether ferroptosis occurs in mammary tissue of dairy goats treated with HC diets. Superoxide dismutase (SOD), catalase (CAT), and glutathione peroxidase (GPx) are key enzymes of the organism’s antioxidant system, and GSH is an important antioxidant, which play a crucial role in the organism’s oxidative and antioxidant homeostasis axis.^28^ It was observed that compared with the LC group, although there was no significant difference in SOD in the mammary gland tissue of dairy goats in the HC group (Figure 4A), CAT activity and GSH content were significantly reduced (Figures 4B and 4C). Furthermore, HC elevated the levels of Fe, Fe^2+^, and malondialdehyde (MDA) in mammary tissues (Figures 4D–4F). Additionally, Western blot analysis demonstrated that HC markedly reduced the expression of ferroptosis-related proteins, including FTH, SLC7A11, and GPX4, while Cox2 expression was elevated (Figures 4G and 4H). Collectively, these findings suggest that HC significantly induces oxidative stress and ferroptosis in mammary tissues.

Changes in the microbiota of the ruminant gastrointestinal tract are often accompanied by changes in microbial metabolism and microbial enzyme activities. Studies have shown that HC diets induce rumen gram-negative bacterial lysis, releasing large quantities of LPS that impair the function of the rumen barrier and cause oxidative stress by allowing the LPS to enter the bloodstream and the liver.^16^^,^^29^ To further investigate the relationship between rumen microbiota, LPS, and ferroptosis, we subsequently assessed oxidative stress and ferroptosis indices in mammary tissues of mice transplanted with rumen microbiota of dairy goats in the LC and HC groups. The results demonstrated that the contents of MDA, Fe, and Fe^2+^ in mammary tissue were elevated in mice from the Abx+RMT^HC^ group in comparison to mice from the Abx and Abx+RMT^LC^ groups (Figures 4M, 4N and 4I). Likewise, the enzymatic activities of the antioxidant indicators CAT and SOD and GSH levels were significantly reduced (Figures 4J–4L). Additionally, elevated serum levels of Fe and Fe^2+^ were observed (Figures 4O and 4P). Similarly, the Western blot results corroborated the reduction in the expression of ferroptosis-related proteins FTH, SLC7A11, and GPX4, while the expression level of Cox2 was increased in mammary tissues of mice with inhibited rumen microbiota in the HC group (Figures 4Q and 4R). In conclusion, our findings indicate that HC induces oxidative stress and ferroptosis in the mammary tissues of dairy goats, which is associated with the dysbiosis of the rumen microbiota and elevated levels of LPS. RMT from dairy goats in the HC group notably induced the development of oxidative stress and ferroptosis in the mammary tissue of mice.

### Fer-1 alleviates mastitis in mice caused by ruminal microbiota transplantation from dairy goats with high concentrations

**Figure 5:**
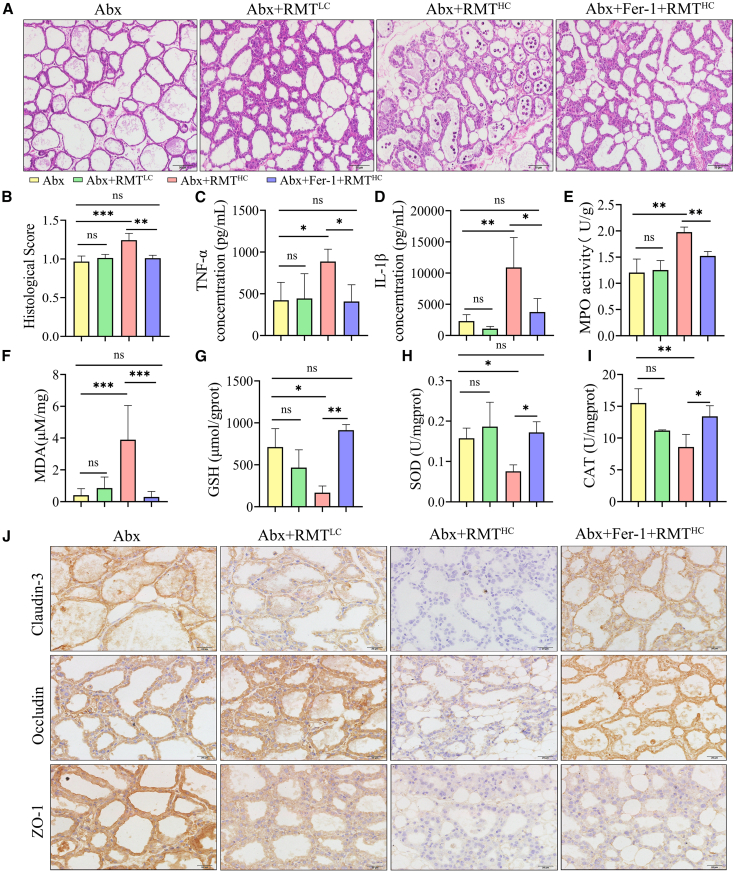
Fer-1 alleviates mastitis in mice caused by RMT from dairy goats with high concentrations (A) Representative H&E images of mammary glands in groups Abx, Abx+RMT^LC^, Abx+RMT^HC^ and Abx+Fer-1+RMT^HC^ (scale bars, 50 μm). (B) Histological score of the mammary gland (*n* = 6). (C–E) Fer-1 significantly reduced Abx+RMT^HC^-induced mammary tissue TNF-α (C) and IL-1β (D) concentrations and MPO activity (E). (F–I) MDA level (F), GSH content (G), and enzyme activities of SOD (H) and CAT (I) in mammary tissues of four groups of mice. (J) Immunohistochemistry was used to determine the localization and expression of TJ proteins in the mammary gland of four groups of mice (scale bars, 20 μm). Data are presented as the means ± SD, and one-way analysis of variance (ANOVA) was performed for statistical analysis. ∗*p* < 0.05, ∗∗*p* < 0.01, ∗∗∗*p* < 0.001, and ∗∗∗∗*p* < 0.0001 indicate significant differences. See also Figure S3.

To further investigate the involvement of ferroptosis in the development of mastitis in the Abx+RMT^HC^ group of mice, we pretreated them with ferrostatin-1 (Fer-1, 1 mg/kg), a ferroptosis inhibitor, via intraperitoneal injection prior to rumen microbiota transplantation from dairy goats. It is noteworthy that in the Abx+Fer-1+RMT^HC^ group, as opposed to the Abx+RMT^HC^ group, mice’ mammary tissue inflammatory cell infiltration decreased and pathohistological scores were reduced (Figures 5A and 5B). Similarly, the concentrations of inflammatory cytokines TNF-α, IL-1β, and MPO activity levels in mammary tissues of mice in the Abx+Fer-1+RMT^HC^ group were reduced in comparison with those in the Abx+RMT^HC^ group and were not significantly different from those of mice in the Abx group (Figures 5C–5E). Additionally, the results demonstrated that Fer-1 pretreatment significantly reduced the MDA level in the mammary tissue of mice transplanted with dairy goat rumen microbiota from the HC group (Figure 5F), while simultaneously increasing the levels of SOD, CAT enzyme activities, and the concentration of GSH (Figures 5G–5I). This was evidenced by the restoration of the expression of the TJs proteins ZO-1, Occludin, and Claudin-3, which was also confirmed by the IHC results (Figure 5J). Furthermore, the Western blot results demonstrated that Fer-1 pretreatment alleviated the disruption of the BMB of the mice’ mammary gland by the rumen microbiota in the transplanted HC group of dairy goats (Figures S3A–S3D). Also, the Western blot results demonstrated that Fer-1 pretreatment significantly restored the expression of ferroptosis-related proteins, including FTH, SLC7A11, and GPX4, while concurrently decreasing the expression of Cox2 protein (Figures S3E–S3I). The findings demonstrate that Fer-1 exerts an inhibitory effect on oxidative stress and ferroptosis in the mammary tissue of mice in the Abx+Fer-1+RMT^HC^ group. Taken together, these results suggest that Fer-1 alleviates mammary tissue inflammation and BMB disruption in mice transplanted with the rumen microbiota of HC group dairy goats by inhibiting oxidative stress and ferroptosis.

### LPS dose-dependently activates ferroptosis to induce inflammation in mouse mammary epithelial cells

Prior research has demonstrated a positive correlation between plasma LPS and oxidative stress indicators, as well as a negative correlation between LPS levels and antioxidant enzyme activity in the liver of HC dairy cows.^16^ In our previous results, we also found that LPS levels in the rumen, mammary gland and plasma of dairy goats were significantly higher in the HC group (Figures 1D, S1B, and S1C). Furthermore, mammary gland and serum LPS levels were also significantly higher in the Abx+RMT^HC^ group of mice (Figures 3C and 3I). Based on these findings, we hypothesize that HC induces a large amount of rumen gram-negative bacterial lysis and LPS release into the bloodstream, which activates oxidative stress and ferroptosis in mammary gland tissues.

**Figure 6:**
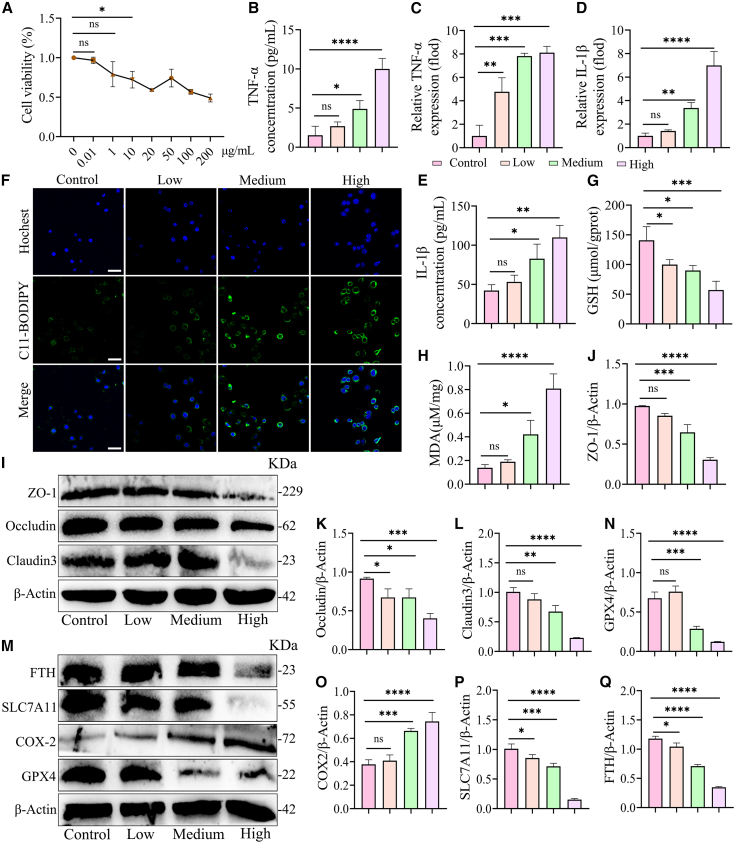
LPS dose-dependently activates ferroptosis to induce inflammation in mouse mammary epithelial cells (A) The impact of different LPS concentrations on MMEC activity was evaluated through a CCK-8 assay. As illustrated in the figure, no notable impact on cellular activity was observed at 1 μM. (B–E) The effects of different doses of LPS on TNF-α (B) and IL-1β (E) in cell supernatant and mRNA levels of TNF-α (C) and IL-1β (D) in cells were investigated. (F) The impact of differing doses of LPS on ROS generation demonstrated that LPS could exert a dose-dependent promotion of ROS generation in MMEC (scale bars, 25 μm). (G) The assay kit detected the GSH content and found that LPS dose dependently reduced the GSH content in MMECs. (H) The assay kit detected MDA levels and found that LPS dose dependently increased MDA levels in MMECs. (I–L) Representative images and relative intensities related to TJ protein expression after low, medium and high doses of LPS acting on MMEC (*n* = 3). (M–Q) Representative images and relative intensities of ferroptosis-associated proteins in MMEC (*n* = 3). Data are presented as the means ± SD, and one-way analysis of variance (ANOVA) was performed for statistical analysis. ∗*p* < 0.05, ∗∗*p* < 0.01, ∗∗∗*p* < 0.001, and ∗∗∗∗*p* < 0.0001 indicate significant differences. See also Figure S4.

The results indicated a positive correlation between intramammary LPS content and mammary tissue MDA levels and Fe^2+^ concentration, and a negative correlation with CAT and SOD enzyme activities and GSH levels in dairy goats in the HC group (Figures S4A–S4E). Likewise, the intramammary LPS content in mice in the Abx+RMT^HC^ group demonstrated a comparable correlation (Figures S4F–S4J). The results indicate that an elevation in intramammary LPS resulting from rumen microbiota dysbiosis may be a pivotal factor in the induction of oxidative stress and ferroptosis. Consequently, the impact of LPS on MMEC was investigated through the CCK-8 assay, which revealed that 10 μg/mL of LPS notably reduced cellular activity and induced ferroptosis in MMEC (Figure 6A). It was also observed that LPS dose-dependently increased the concentration and mRNA levels of TNF-α and IL-1β in MMEC (Figures 6B–6E). As illustrated in Figure 6F, the ROS levels in MMEC cells exhibited a dose-dependent elevation in response to LPS. Similarly, LPS dose-dependently decreased GSH concentrations and increased MDA levels (Figures 6G and 6H). Similarly, the Western blot experiments demonstrated that LPS dose-dependently decreased the expression of TJs protein in MMEC (Figures 6I–6L). The expression of ferroptosis -related proteins, including FTH, SLC7A11, and GPX4, also exhibited a gradual decrease with increasing LPS concentration, accompanied by a corresponding increase in COX-2 protein expression (Figures 6M and 6Q). Collectively, these findings suggest that LPS dose-dependently induces oxidative stress and activates ferroptosis in MMEC.

### Fer-1 alleviates LPS-induced inflammation in mouse mammary epithelial cells by inhibiting ferroptosis

**Figure 7:**
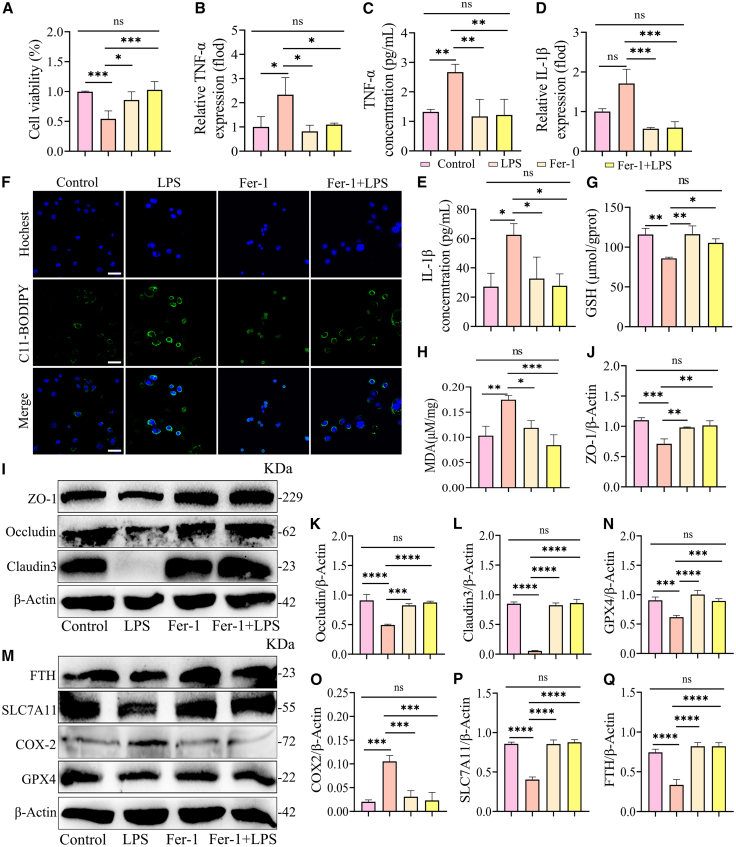
Fer-1 alleviates LPS-induced inflammation in mouse mammary epithelial cells by inhibiting ferroptosis (A) The effect of Fer-1 pretreatment followed by LPS stimulation on MMEC activity was assessed by the CCK-8 assay. (B–E) The effects of Fer-1 pretreatment on TNF-α (B) and IL-1β (E) in cell supernatants and TNF-α (C) and IL-1β (D) mRNA levels in cells were investigated. (F) Fer-1 significantly inhibited LPS-induced ROS accumulation in MMEC (scale bars, 25 μm). (G) GSH content. (H) MDA level. (I–L) Fer-1 pretreatment significantly restored TJ protein expression in LPS-induced MMEC (*n* = 3). (M–Q) Representative images and relative intensities of ferroptosis-associated proteins in MMEC after Fer-1 preprocessing (*n* = 3). Data are presented as the means ± SD, and one-way analysis of variance (ANOVA) was performed for statistical analysis. ∗*p* < 0.05, ∗∗*p* < 0.01, ∗∗∗*p* < 0.001, and ∗∗∗∗*p* < 0.0001 indicate significant differences.

To further elucidate the contribution of ferroptosis to LPS-induced MMEC inflammation, we pretreated MMEC with Fer-1 (10 μM). The results demonstrated that Fer-1 considerably inhibited LPS-induced MMEC death (Figure 7A). In accordance with our hypothesis, Fer-1 exhibited a pronounced inhibitory effect on the LPS-induced elevation in TNF-α and IL-1β concentrations and mRNA levels in MMEC (Figures 7B–7E), indicating that Fer-1 attenuated LPS-induced MMEC inflammation to a certain extent. Of particular note is the observation that Fer-1 effectively suppressed ROS levels in MMEC (Figure 7F), indicating that Fer-1 may have attenuated LPS-induced oxidative damage in MMEC. Furthermore, it was observed that Fer-1 restored GSH levels and reduced MDA concentrations (Figures 7G and 7H). It was also observed that Fer-1 resulted in a notable restoration of the expression of TJ proteins, including ZO-1, Occludin, and Claudin-3 in MMEC compared to the LPS group (Figures 7I–7L). Similarly, Fer-1 restored the expression of ferroptosis-associated proteins FTH, SLC7A11, and GPX4 and reduced the expression of COX-2 in comparison to the LPS group (Figures 7M–7Q). Collectively, these results indicate that Fer-1 alleviates MMEC inflammation by inhibiting LPS-induced oxidative stress and ferroptosis.

## Discussion

The dairy industry is a signature industry of agricultural modernity, and its sound growth depends not only to the interests of dairy farmers but also on the public’s health and the safety of dairy products.^30^ Mastitis, on the other hand, is one of the most devastating diseases that harms the health, performance, and reproductive potential of dairy cows and dairy goats, severely limiting the dairy industry’s expansion and resulting in massive global economic losses.^31^ Although pathogenic microbial infections are the primary causative agent of mastitis, there is accumulating evidence that rumen microbiota dysbiosis contributes to the development of mastitis in ruminants.^32^ The hypothesis of a rumen-mammary axis has been proposed, but the underlying mechanisms remain to be elucidated. In clinical practice, ruminants are frequently provided with excessive HC diets with the objective of rapidly enhancing growth and lactation performance, which in turn results in the development of a range of metabolic diseases, including SARA.^10^ Additionally, the rumen, the largest and most important digestive organ of ruminants, contains a vast number of microorganisms. The rumen microbiota plays a pivotal role in various host functions, including absorption, digestion, metabolism, immune homeostasis, and neuroendocrine regulation.^33^ Recent studies have increasingly demonstrated that SARA significantly reduces the abundance and diversity of rumen microbiota in dairy cows and dairy goats.^34^ Concurrently, SARA dairy cows exhibited elevated levels of the *Firmicutes* and *Actinobacteria* within the rumen, accompanied by a reduction in the abundance of *Rickettsiales*, *Acholeplasmatales*, *Victivallaceae*, *Sutterella*, and *Shuttleworthia*.^35^ Oxidative stress is linked to a number of disease processes in ruminants. Furthermore, LPS released from rumen gram-negative bacteria that undergo lysis during the occurrence of SARA crosses the damaged rumen barrier and migrates with the bloodstream to the mammary gland, where it induces oxidative stress in mammary tissues.^16^ It is worthy of note that oxidative stress and ferroptosis are closely related, with ferroptosis playing a significant role in the development of mastitis. Nevertheless, no study has hitherto reported whether ferroptosis is involved in the development of mastitis during SARA.

The imbalance between ROS production and antioxidant defense capacity within the organism represents the primary causal factor in induced oxidative stress.^14^ Normally, two antioxidant defense systems are present within an organism: an enzymatic system and a non-enzymatic system. The enzymatic system is primarily composed of autogenous antioxidant enzymes, including SOD, CAT, and GSH-Px, while the non-enzymatic antioxidant defense system is constituted by substances such as GSH, vitamin C, and melatonin.^36^ Previous studies have demonstrated that HC diets have been associated with a reduction in antioxidant enzyme activities within mammary tissues and blood, a decline in GSH levels and an overall weakening of the total antioxidant capacity of mammary tissues.^27^ It was also observed that long-term feeding of HC diets resulted in impaired antioxidant defenses, oxidative stress, and liver damage in dairy cows.^37^ It is noteworthy that ferroptosis, a form of cell death resulting from oxidative damage to cells caused by the accumulation of intracellular iron ions, is dependent on iron and lipid peroxidation.^17^ Additionally, it can be induced by two antagonistic processes: intracellular lipid peroxide production and elimination. The molecular basis of ferroptosis encompasses glutamate/cystine transporter proteins, the antioxidant system (GPX4), iron metabolism, unsaturated fatty acid metabolism, and the ferroptosis inhibitory protein 1 ubiquinone system.^19^ There is a close correlation between oxidative stress and ferroptosis, as evidenced by the fact that several major regulatory pathways of ferroptosis are associated with oxidative stress and ROS overproduction.

The present study demonstrated that feeding an HC diet for a period of eight weeks resulted in a significant reduction in rumen pH, leading to the development of SARA. These findings are in accordance with those of previous studies, which have also shown that HC diet feeding significantly reduced the expression and distribution of mammary TJs proteins in dairy goats, thereby damaging the BMB and inducing mastitis. Furthermore, the occurrence of SARA was accompanied by a notable reduction in the rumen microbiota diversity, accompanied by significant alterations in its composition and function. Among the most notable changes were a pronounced decline in the abundance of *Rikenellaceae_RC9_gut_group* and *norank_f Eubacterium_coprostanoligenes_group*, while the abundance of *norank_f_Bifidobacteriaceae* exhibited a marked increase. Notably, our study demonstrated that RMT from the HC group, but not the LC group, resulted in the occurrence of mouse mastitis. This was associated with an increase in LPS-activated ferroptosis in the mammary gland. This hypothesis was corroborated by the alleviation of mammary gland inflammation and the disruption of the BMB in mice following the use of the ferroptosis inhibitor Fer-1. Moreover, cellular experiments corroborated the assertion that ferroptosis plays a pivotal role in LPS-induced inflammation in MMEC. Specifically, prolonged feeding of HC diets resulted in a reduction in rumen pH, lysis of gram-negative bacteria, and the release of large amounts of LPS into the bloodstream. This subsequently led to the transportation of LPS to the mammary gland, where it decreased the total antioxidant capacity of mammary tissues, activated ferroptosis, and induced mastitis.

Within the intricate regulatory network of mastitis, ferroptosis emerges as a pivotal consequence of uncontrolled inflammation and oxidative stress, functioning as a central catalyst in the propagation of tissue damage. It has been demonstrated by preceding studies that in the event of pathogens breaching the BMB, their pattern recognition molecules activate Toll-like receptors. This activation subsequently triggers NF-κB-mediated explosive inflammatory responses and NADPH oxidase-dependent ROS bursts.^38^ These, in turn, trigger ferroptosis. On the one hand, inflammatory signals result in the abnormal accumulation of intracellular free iron pools. This is achieved by means of the upregulation of iron transporters and the reduction of iron storage proteins. Conversely, sustained ROS stress has been shown to exhaust GSH and inhibit the activity and expression of key antioxidant enzyme GPX4. Furthermore, iron overload and lipid peroxidation have been demonstrated to result in cell ferroptosis. It is important to note that ferroptosis cells release a significant number of molecular patterns and lipid peroxides that are associated with damage. These further activate cellular inflammasomes and expand the inflammatory cascade, exacerbating the inflammatory response.^39^ In the present study, the hypothesis that the targeted inhibition of ferroptosis has therapeutic potential in the treatment of mastitis was investigated. The findings of Bao et al. that targeted inhibition of ferroptosis can alleviate Staphylococcus aureus-induced mastitis in mice and reduce the levels of pro-inflammatory cytokines TNF-α and IL-6 in the mammary gland were corroborated.^40^ It is hypothesized that this process may be related to the prevention of the propagation of membrane damage and the inhibition of the inflammatory cycle. Despite the demonstrated efficacy of ferroptosis inhibitors in the treatment of mastitis, their clinical application remains encumbered by numerous challenges.

### Limitations of the study

This study revealed the innovative mechanism by which a high-precision diet induces rumen microbiota disruption and LPS elevation in dairy goats, triggering ferritinophagy-ferroptosis leading to mastitis. However, there are still several important limitations. Firstly, it is evident that RMT is unable to fully replicate the complex *in situ* microenvironment of the rumen in ruminants. Secondly, the blank state of the microbiota caused by antibiotic pretreatment itself may exacerbate immune disorders or metabolic abnormalities, thereby amplifying the effects of LPS. Then, the research focuses on ferroptosis as a terminal cell death pathway, but does not fully exclude the parallel contributions of other cell death pathways, such as apoptosis and necroptosis. LPS, as a key trigger factor, may have a synergistic effect *in vivo* with other rumen-derived metabolites, such as histamine, ethanol and short-chain fatty acids. These limitations indicate that, in the future, it will be necessary to combine *in situ* rumen perfusion, multi omics time-series analysis, conditional gene knockout animal models, and clinical intervention trials in order to more comprehensively and rigorously validate this pathway and promote the exploration of its application.

## Resource availability

### Lead contact

Further information and requests for resources and reagents should be directed to and will be fulfilled by the lead contact, Yue Zhang (zhangyue888@jlnku.edu.cn).

### Materials availability

This study did not generate new unique reagents.

### Data and code availability

16S rRNA sequencing data for all samples have been deposited in NCBI and are publicly available as of the date of publication (NCBI Sequence Read Archive [SRA] repository: PRJNA1337779). Accession numbers are also listed in the key resources table. Original western blot images and microscopy data reported in this article will be shared by the lead contact upon request.

This study did not generate any unique code.

Any additional information required to reanalyze the data reported in this article is available from the lead contact upon request.

## Acknowledgments

We would like to express our heartfelt gratitude to Yushan Liu from Zhang’s laboratory for their invaluable contributions to this study. The study was supported by the National Key Research and Development Program of China (2023YFD1801101).

## Author contributions

Y.H. and W.Z. designed the study. Y.H. wrote the article. Y.H. and C.Z. performed all mouse animal experiments and all statistical analyses. H.Y., C.Z., and N.S. assisted with animal experiments. Z.Z., P.C., and Y.C. completed the cell experiments. Y.F. reviewed the article. X.H. and Y.Z. obtained funding. All authors revised and approved the article.

## Declaration of interests

The authors declare that they have no known competing financial interests or personal relationships that could appear to influence the work reported in this article.

## STAR★Methods

### Key resources table

**Table:** 

REAGENT or RESOURCE	SOURCE	IDENTIFIER
**Antibodies**		

Anti-FTH	Affinity Biosciences	#DF6278; RRID: AB_2838244
Anti-SLC7A11	Affinity Biosciences	#DF12509; RRID: AB_2845314
Anti-Cox2	Affinity Biosciences	#AF7003; RRID: AB_2835311
Anti-GPX4	Affinity Biosciences	#DF6701; RRID: AB_2838663
Anti-Occludin	Affinity Biosciences	#DF7504; RRID: AB_2841004
Anti-ZO-1	Affinity Biosciences	#AF5145; RRID: AB_2837631
Anti-Claudin-3	Affinity Biosciences	#AF0129; RRID: AB_2833313
Anti-β-actin	Affinity Biosciences	#AF7018; RRID: AB_2839420

**Chemicals, peptides, and recombinant proteins**		

Ampicillin	Sigma	Cat# A5354
Neomycin	Sigma	Cat# N6386
Metronidazole	Sigma	Cat# 16677
Vancomycin	Sigma	Cat# V2002
Ferrostatin-1	Sigma	SML0583
LPS	Absin	Abs42020800
TRIzol Reagent	Invitrogen	Cat# 15596026
C11-BODIPY 581/591	Sigma	SML3717

**Critical commercial assays**		

Mouse TNF-α ELISA assay kit	Biolegend	Cat. No. 430915
Mouse IL-1β ELISA assay kit	Biolegend	Cat. No.432615
Myeloperoxidase assay kit	Lanpaibio	hj-C1253
SAP (Mouse/Rabbit) IHC Kit	Maixin Biotechnology	KIT-7710
TransStart Tip Green qPCR SuperMix	TransGen Biotech	AQ141-01
FastStart Universal SYBR Green Master Mix	Roche	4913850001

**Deposited data**		

16S rRNA MiSeq data	NCBI BioProject	PRJNA1337779

**Experimental models: Organisms/strains**		

BALB/c	Liaoning Changsheng biotecnology co., Ltd.	–

**Oligonucleotides**		

See Table S1 for primers	–	–

**Software and algorithms**		

Prism v8.0c	GraphPad Software	https://www.graphpad.com/scientificsoftware/prism/
ImageJ	ImageJ	https://imagej.net/software/imagej/
Data are presented as the means ± SD and one-way analysis of variance (ANOVA) was performed for statistical analysis.	GraphPad Software	∗*p* < 0.05, ∗∗*p* < 0.01, ∗∗∗*p* < 0.001 and ∗∗∗∗*p* < 0.0001 indicate significant differences.

### Experimental model and study participant details

Approval for all experiments involving animals was granted by the Institutional Animal Care and Use Committee (IACUC) of Jilin University, with approval number 2023-089. The IACUC ethics committee conducted a comprehensive review of the proposal and subsequently granted the requisite animal care and use permit license. In conducting all experiments, we adhered to the guidelines set forth in the US National Institutes of Health’s manual of animal laboratory care and use.

In this study, 12 dairy goats (2–3 years old, average weight 55 kg) were obtained from a farm in Changchun, Jilin Province. All of the goats had no history of disease or medication within the six months preceding the commencement of the study. A random division of the dairy goats was conducted into two groups: the low concentrate (LC) group and the high concentrate (HC) group. Both groups met their daily nutritional requirements. The LC group was fed a standard diet, while the HC group was fed HGD (30% forage and 70% grain) for 8 weeks. SARA was diagnosed based on rumen pH, and rumen fluid was collected from the dairy goats as per previous studies.^41^ Precisely, following a 6-h period of feeding on consecutive mornings, the collection of rumen fluid is initiated once per hour. Approximately 100 mL of rumen fluid is collected on each occasion, and is then immediately filtered through four layers of sterile gauze. Following filtration, the pH of the filtrate is measured immediately using a portable pH meter. Subsequent to this, 50 mL of the filtered rumen fluid is aliquoted, expeditiously transferred to a sample tube, and immediately stored in a −80°C refrigerator for the purpose of subsequent analysis of rumen microbial community composition.

In the mouse experiment, SPF BABL/c mice (57 females and 19 males) aged 6–8 weeks and weighing 23–25 g were supplied by Liaoning Changsheng Biotechnology Co., Ltd. All mice were provided with unrestricted access to water and food. Female mice were housed with male mice in a ratio of three females to one male until the onset of pregnancy, after which the male mice were removed. The mice were divided into four groups: (1) Abx group: mice were gavaged with antibiotics. (2) Abx+RMT^LC^ group: RMT from LC group of dairy goats. (3) Abx+RMT^HC^ group: RMT from HC group of dairy goats. (4) Abx+Fer-1+RMT^HC^ group: mice were pre-treated with Fer-1 (1 mg/kg) by intraperitoneal injection half an hour before transplantation of the HC group of dairy goat rumen microbiota. The particular methodologies employed in RMT were consistent with those utilized previously.^42^ Prior to RMT procedures, all gravid murine subjects underwent a 5-day antibiotic pretreatment regimen (ampicillin 200 mg/kg, vancomycin 100 mg/kg, neomycin 200 mg/kg, metronidazole 200 mg/kg) to achieve intestinal commensal microbiota depletion.^43^ Microbial reconstitution was accomplished through oral gavage of 300 μL sterile-filtered donor rumen fluid supernatant administered consecutively for 3 days, followed by biweekly administrations maintained throughout the 21-day gestational observation period.

MMEC (HC11 cell) were obtained from the American Type Culture Collection (ATCC, CRL3062) and cultured in a DMEM supplemented with 10% fetal bovine serum and 1% ampicillin and streptomycin at 37°C with 5% CO2. The impact of LPS on the viability of MMEC cells was investigated through the CCK8 assay. In the ferroptosis inhibition assay, cells were pre-treated with Fer-1 (10 μM) for a period of 6 h prior to LPS stimulation.

### Method details

#### Cell viability assay

Cellular viability assessments of MMECs post-LPS challenge were conducted employing a CCK8(CA1210, Solarbio, China) following manufacturer’s protocol.

#### MDA/GSH/iron assay

MDA levels in mammary tissue (10% tissue homogenate) and MMEC (1×10^6^ cells) lysates were quantified via thiobarbituric acid reactive substances assay (Jiancheng Biotech). GSH content in biological specimens was determined enzymatically using glutathione reductase-coupled 5,5′-dithiobis(2-nitrobenzoic acid) (DTNB) oxidation (Jiancheng Biotech). Total iron concentration in mammary parenchyma was assessed via bathophenanthroline disulfonate chelation methodology (Jiancheng Biotech).

#### Measurements of enzymes

SOD activity was determined through xanthine oxidase-mediated nitroblue tetrazolium (NBT) reduction inhibition, while CAT activity was quantified via ammonium molybdate colorimetry (Jiancheng Biotech).

#### Lipid ROS assay

MMEC were loaded with C11-BODIPY 581/591 (10 μM) for a period of 30 min, followed by three PBS washes (5 min each). ROS generation was visualized using confocal microscopy (FV3000, Olympus) with dual-excitation (488/594 nm) and emission capture (510–550 nm/590–650 nm).

#### 16S rRNA

Genomic DNA isolation from rumen liquid specimens was performed employing the E.Z.N.A. Soil DNA Kit with mechanical lysis and silica-membrane purification. Nucleic acid integrity was verified through electrophoretic assessment, while quantification was conducted via UV spectrophotometry. Following this, the DNA was diluted to 1 ng/μL with sterile water. The purified DNA concentrations for 16S rRNA and metagenomic sequencing were measured using NanoDrop2000. The purified PCR products were subjected to library construction using the NEXTFLEX Rapid DNA-Seq Kit and sequenced using the Illumina PE300/PE250 platform. The quality of the bipartite raw sequencing sequences was assessed using Fastp software, and FLASH software was employed for splicing. Subsequently, the sequences resulting from the QC splicing were subjected to Operational Taxonomic Unit (OTU) clustering, and chimeric sequences were excluded based on a similarity threshold of 97% using the UPARSE software. Subsequently, OTU species taxonomic annotation was conducted in comparison with the Silva 16S rRNA gene database, with a confidence threshold of 70%. Additionally, the community composition of each sample was quantified at varying species classification levels.

#### Histological analysis

All mammary tissue samples were fixed in 4% paraformaldehyde for 48 h prior to processing into paraffin-embedded sections. Hematoxylin-eosin (H&E) alone was then used to stain these sections, and pathological abnormalities such as inflammatory cell infiltration and structural alterations to the mammary gland were later noted. Inflammation scores were determined as follows for all samples, as previously mentioned: 0, none; 1, mild; 2, moderate; 3, severe.^44^

#### Determination of inflammatory cytokines by ELISA

Mammary gland tissue weighing 0.03 g was collected and homogenized at a ratio of 1:9 using cold PBS in a tissue homogenizer. The tissue was homogenized and centrifuged for 10 min at 12000 rpm and 4°C to measure the levels of TNF-α and IL-1β.

#### LPS determination

The separation of serum samples by centrifugation (12,000×g, 4°C, 10 min) was conducted prior to the detection of cytokines and LPS using ELISA kits. The kits utilized were those designed for dairy goats and mice, respectively, and were employed in accordance with the instructions provided by the manufacturer (Lanpaibio Shanghai, China).

#### MPO activity

Mammary tissue was procured, a 10% tissue homogenate was prepared, and the MPO kit was used to quantify the MPO levels in the mammary tissue.

#### Western blot

The BCA kit was used to assess the protein concentrations after the total mammary proteins were extracted using the Tissue Protein Extraction Kit. Subsequently, equivalent quantities (50 μg) of protein were transferred to PVDF membranes that had undergone either 10% or 12% SDS-PAGE. Following a 3-h incubation period at room temperature with 5% skimmed milk, the PVDF membranes were incubated overnight at 4°C with a 1000-fold dilution of the specific antibody. On the subsequent day, the PVDF membranes were rinsed three times for 20 min each time with TBST. Subsequently, the membranes were incubated for 2 h at room temperature with either a goat anti-rabbit secondary antibody (Affinity, #S0001, 1:20000) or a goat anti-mouse secondary antibody (Affinity, #S0002, 1:20000). Subsequently, the signals present on the PVDF membranes were analyzed using the ECL detection system. Protein loading was normalized to β-actin, which served as the internal reference.

#### Immunohistochemistry (IHC)

The mammary sections were dewaxed using xylene and subsequently dehydrated in a graded alcohol series. Subsequently, antigen recovery was conducted using sodium citrate, followed by treatment with a per-endogenous oxidase blocker (SAP (Mouse/Rabbit) IHC Kit, MXB, China) for 40 min at room temperature. Following this, the sections were washed on three occasions, with a 5-min interval between each wash, using PBS. Tissue samples were incubated in normal non-immune goat serum (SAP (Mouse/Rabbit) IHC Kit, MXB, China) for 40 min at room temperature.

Tissue sections underwent sequential incubations with primary antibodies at 4°C for 16–18 h, followed by secondary antibodies (goat anti-rabbit IgG) for 30 min. Subsequently, the samples were incubated with horseradish peroxidase (HRP) for a period of 20 min. Following three washes with PBS for 5 min each, the sections were rinsed for 3 min using a chromogenic agent. Next, the sections were observed under a microscope and terminated with water based on color. After a final hematoxylin staining for 5 min, the sections were treated with the 1% hydrochloric acid-ethanol differential method and ammonium hydroxide. Following dehydration, the sections were mounted with neutral resin.

#### Quantitative real-time PCR

Cell samples were obtained and total RNA extracted using the Trizol reagent. Reverse transcription of cDNA was conducted using a TransStart Tip Green qPCR SuperMix and specific primers were used with a FastStart Universal SYBR Green Master Mix (ROX) (Roche, Switzerland, Basel) in a Step One Plus apparatus. The specific primers employed in this study are presented in Table S1. To ensure the reliability of the results, GAPDH was utilized as an endogenous control for the cells, and the 2^−ΔΔCt^ method was implemented to calculate the relative expression of genes, with the control group serving as the calibrator.

#### Statistical analysis

The statistical analysis of the experimental data was conducted using GraphPad Prism 8.0 (Manufacturer, La Jolla, CA, USA). To ascertain significant differences between two groups, the Mann-Whitney *U* test (non-parametric) or two-tailed unpaired Student’s *t* test (parametric) were employed. Furthermore, ANOVA was employed to compare three or more groups. Tukey’s test was applied as a post hoc test for parametric samples. *p* < 0.05 or *p* < 0.01 was considered statistically significant.
