# Supplementary material for: Ruminal microbial dysbiosis induces mastitis in dairy goats by activating oxidative stress and ferritinophagy-ferroptosis
Source: iScience. 2025 Nov 12;28(12):114012. doi: 10.1016/j.isci.2025.114012 (PMC12682052; doi:10.1016/j.isci.2025.114012)
Supplement: Document S1. Figures S1–S4 and Table S1 [file mmc1.pdf]

iScience, Volume *28*

Supplemental information

Ruminal microbial dysbiosis induces mastitis in dairy goats by activating oxidative stress and ferritinophagy-ferroptosis

Yuhong He, Wei Zhang, Can Zhang, Nier Su, Zeming Zhou, Chong Peng, Chongshan Yuan, Yunhe Fu, Xiaoyu Hu, and Yue Zhang

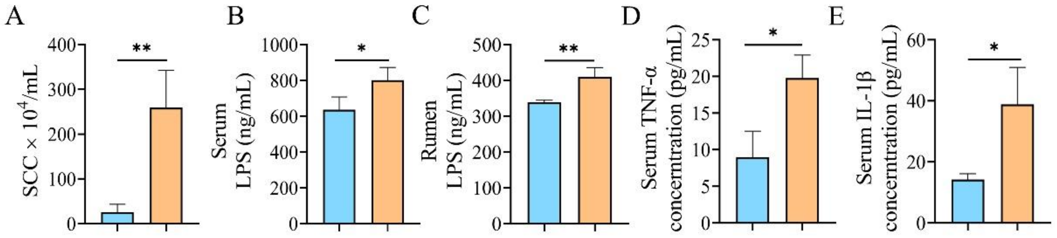

F

Characteristics	LC	HC	P-value
ALB (g/L)	27.16 ± 3.92	29.86 ± 4.42	0.3879
TP (g/L)	70.16 ± 5.59	70.78 ± 6.70	0.8905
GLOB (g/L)	43.00 ± 5.61	40.90 ± 7.12	0.6554
A/G	0.65 ± 0.13	0.76 ± 0.21	0.3661
ALT (U/L)	26.40 ± 14.35	22.00 ± 3.58	0.5682
ALP (U/L)	119.60 ± 53.74	199.00 ± 95.59	0.2360
SCR (μM)	54.60 ± 10.31	74.30 ± 7.65	0.0154
BUN (mM)	5.67 ± 1.58	7.68 ± 1.69	0.1206
S/B	104.59 ± 25.09	103.54 ± 19.99	0.9494
Glu (mM)	3.51 ± 0.54	3.64 ± 0.30	0.6906

G

Characteristics	LC	HC	P-value
WBC (10^9/L)	9.03 ± 2.71	14.43 ± 5.08	0.0569
Neu %	3.10 ± 0.52	7.88 ± 4.17	0.0399
Lym %	5.17 ± 1.74	4.93 ± 1.59	0.8503
Mon %	0.81 ± 0.49	0.52 ± 0.17	0.4274
Eos %	0.16 ± 0.10	0.30 ± 0.08	0.0968
Bas %	0.00 ± 0.00	0.03 ± 0.04	0.2248

Figure S1. High-concentrate diet induces mastitis in dairy goats. Related to Figure 1.

(A) Number of somatic cells in milk of dairy goats in LC and HC groups (n=6).

(B) Serum LPS content of dairy goats in LC and HC groups (n=6).

(C) Rumen LPS content of dairy goats in LC and HC groups (n=6).

(D-E) Concentrations of serum TNF-α (D) and IL-1β (E) in dairy goats of LC and HC groups (n=6).

(F) Biochemical characteristics of blood in dairy goats.

(G) Hematological characteristics of dairy goat blood.

Data are presented as the means ± SD and one-way analysis of variance (ANOVA) was performed for statistical analysis. *$p < 0.05$, **$p < 0.01$, *** $p < 0.001$ and **** $p < 0.0001$ indicate significant differences.

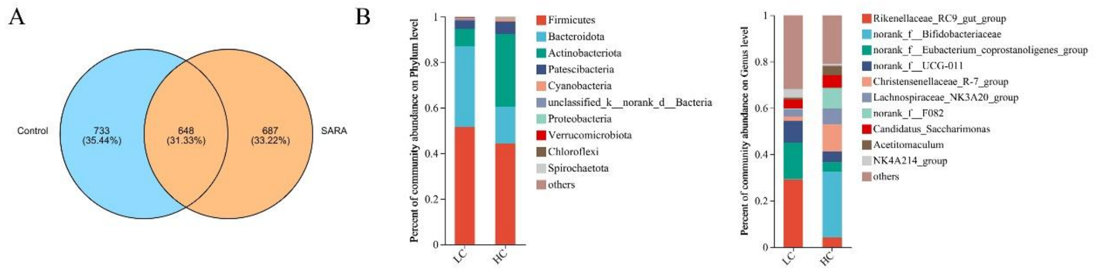

Figure S2. High-concentrate diets induce rumen microbiota dysbiosis in dairy goats. Related to Figure 2.

(A) Venn diagram results showed that rumen microbiota composition was different between LC and HC groups of dairy goats.

(B) Composition of rumen microbiota at the phylum level in dairy goats of LC and HC groups.

(C) Composition of rumen microbiota at the genus level in dairy goats of LC and HC groups.

Data are presented as the means ± SD and one-way analysis of variance (ANOVA) was performed for statistical analysis. $*p < 0.05$, $**p < 0.01$, $***\ p < 0.001$ and $****\ p < 0.0001$ indicate significant differences.

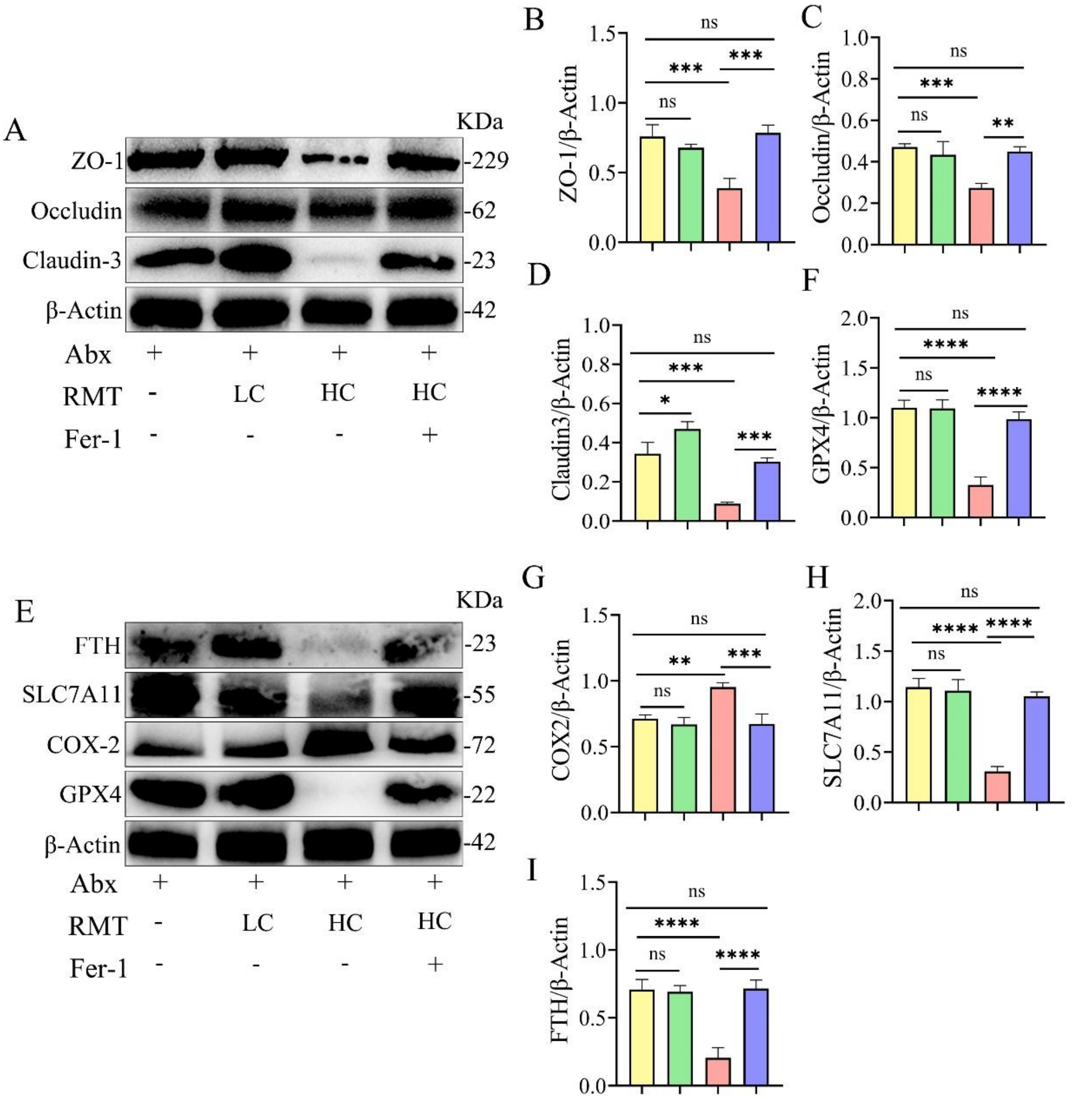

Figure S3. Fer-1 alleviates mastitis in mice caused by RMT from dairy goats with high concentrations. Related to Figure 5.

(A-D) Representative images and relative intensities related to TJs proteins in mammary tissues of groups Abx, Abx+RMT^{LC}, Abx+RMT^{HC} and Abx+Fer-1+RMT^{HC}.

(E-I) Representative images and relative intensities related to ferroptosis-associated proteins in mammary tissues of groups Abx, Abx+RMT^{LC}, Abx+RMT^{HC} and Abx+Fer-1+RMT^{HC}.

Data are presented as the means ± SD and one-way analysis of variance (ANOVA) was performed for statistical analysis. *p < 0.05, **p < 0.01, *** p < 0.001 and **** p < 0.0001 indicate significant differences.

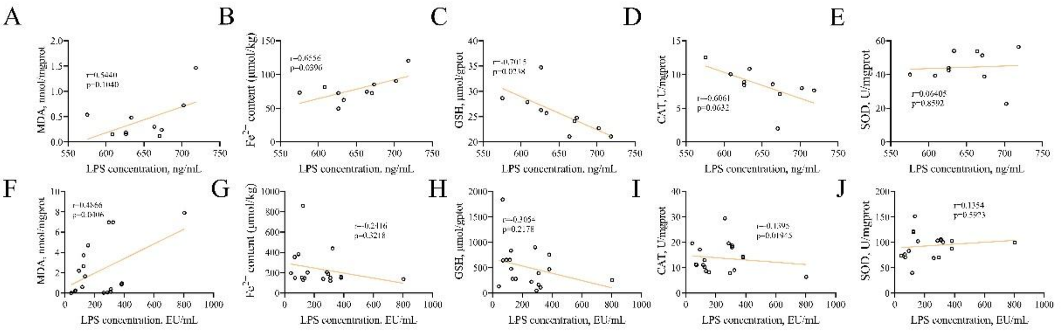

Figure S4. Fer-1 alleviates LPS-induced inflammation in mouse mammary epithelial cells by inhibiting ferroptosis. Related to Figure 7.

(A) Correlation analysis between serum LPS concentration and mammary tissue MDA level in dairy goats of HC group.

(B) Correlation analysis between serum LPS concentration and mammary tissue Fe^{2+} concentration in dairy goats of HC group.

(C) Correlation analysis between serum LPS concentration and mammary tissue GSH level in dairy goats of HC group.

(D) Correlation analysis between serum LPS concentration and mammary tissue enzymatic activity of CAT in dairy goats of HC group.

(E) Correlation analysis between serum LPS concentration and mammary tissue enzymatic activity of SOD in dairy goats of HC group.

(F) Correlation analysis between serum LPS concentration and MDA level in Abx+RMT^{HC} group of mice.

(G) Correlation analysis between serum LPS concentration and Fe^{2+} concentration in Abx+RMT^{HC} group of mice.

(H) Correlation analysis between serum LPS concentration and GSH level in Abx+RMT^{HC} group of mice.

(I) Correlation analysis between serum LPS concentration and enzymatic activity of CAT in Abx+RMT^{HC} group of mice.

(J) Correlation analysis between serum LPS concentration and enzymatic activity of SOD in Abx+RMT^{HC} group of mice.

Table S1 Primers used in this study.

Gene	Primer	Sequence(5' to 3')
GADPH	sence	AGGTCGGTGTGAACGGATTTG
	antisence	TGTAGACCATGTAGTTGAGGTCA
TNF-α	sence	CCCTCACACTCAGATCATCTTCT
	antisence	GCTACGACGTGGGCTACAG
IL-1β	sence	GCAACTGTTCCTGAACTCAACT
	antisence	ATCTTTTGGGGTCCGTCAACT
